# Potential Retinoid X Receptor Agonists for Treating Alzheimer's Disease from Traditional Chinese Medicine

**DOI:** 10.1155/2014/278493

**Published:** 2014-04-30

**Authors:** Kuan-Chung Chen, Yu-Cheng Liu, Cheng-Chun Lee, Calvin Yu-Chian Chen

**Affiliations:** ^1^School of Pharmacy, China Medical University, Taichung 40402, Taiwan; ^2^School of Medicine, College of Medicine, China Medical University, Taichung 40402, Taiwan; ^3^Department of Biomedical Informatics, Asia University, Taichung 41354, Taiwan

## Abstract

Alzheimer's disease is neurodegenerative disorder due to the accumulation of amyloid-**β** in the brain and causes dementia with ageing. Some researches indicate that the RXR agonist, Targretin, has also been used for treatment of Alzheimer's disease in mouse models. We investigate the potent candidates as RXR agonists from the vast repertoire of TCM compounds in TCM Database@Taiwan. The potential TCM compounds, **β**-lipoic acid and sulfanilic acid, had higher potent binding affinities than both 9-*cis*-retinoic acid and Targretin in docking simulation and have stable H-bonds with residues Arg316 and some equivalent hydrophobic contacts with residues Ala272, Gln275, Leu309, Phe313, Val342, Ile345, and Cys432 as Targretin. The carboxyl or sulfonyl hydroxide group can form a H-bond with key residue Arg316 in the docking pose, and the phenyl group next to the carboxyl or sulfonyl hydroxide group can form a **π** interaction with residue Phe313. Moreover, **β**-lipoic acid and sulfanilic acid have stable H-bonds with residue Gln275, Ser313, and residue Ala327, respectively, which may strengthen and stabilize TCM candidates inside the binding domain of RXR protein. Hence, we propose **β**-lipoic acid and sulfanilic acid as potential lead compounds for further study in drug development process with the RXR protein against Alzheimer's disease.

## 1. Introduction

Alzheimer's disease is serious problem which will cause huge cost for taking care of the patient. It is neurodegenerative disorder due to the accumulation of amyloid-*β* in the brain and causes dementia with ageing [1, 2]. The cholesterol transport protein apolipoprotein E plays the important role in the clearance of amyloid-*β* from the brain [3]. In addition, the transcription of apolipoprotein E expression is also regulated by the heterodimers of RXR with PPAR-*γ* and LXRs [4, 5]. The RXR agonist, Targretin, has also been used for treatment of Alzheimer's disease in mouse models [6].

The retinoic X receptors (RXRs) belong to a superfamily of eukaryotic transcription factors. They are ligand-dependent nuclear receptors involved in the processes of retinoid signaling in normal hematopoiesis [7, 8] and cell development such as cell patterning, organogenesis, proliferation, and differentiation [9, 10]. RXRs have three different isoforms (*α*, *β*, and *γ*) which form heterodimers with other nuclear receptors, such as retinoid acid receptor (RAR), peroxisome proliferator-activated receptor (PPAR), farnesoid X receptor (FXR), liver X receptor (LXR), thyroid hormone receptor (TR), and vitamin D receptor (VDR) as coregulators [11–17]. The transcription of RXRs is activated by endogenous 9-*cis*-retinoic acid, which is a vitamin A derivative for regulation of growth and morphogenesis [18–21]. The selective synthetic ligands for the RXRs have been also indicated as therapeutic agents to treat cancer and dermatological diseases [22–27].

Nowadays, more and more mechanisms of diseases had been determined to detect the useful target protein against diseases [28–33]. Previously to* in silico* drug discovery researches, many compounds extracted from traditional Chinese medicine (TCM) have been indicated as potential lead compounds used for wide range of diseases, including metabolic syndrome [34–36], stroke [37–40], cancers [41–45], influenza [46–49], viral infection [50], diabetes [51], inflammation [52], and some other diseases [53, 54]. To improve drug discovery from TCM compounds, we aim to investigate the potent candidates as RXR agonists from the vast repertoire of TCM compounds in TCM Database@Taiwan. As the side effect and ligand binding with target protein may affect by the structural disorders of residues in the protein [55, 56], the prediction of disordered amino acids of RXR protein was performed before virtual screening. After virtual screening the TCM compounds, the molecular dynamics (MD) simulations were then employed to study protein dynamics and analyze the stability of interactions for each docking poses of TCM candidates.

## 2. Materials and Methods

### 2.1. Data Collection

The X-ray crystallography structure of the intact PPAR-*γ*-RXR-nuclear receptor complex on DNA with 9-*cis*-retinoic acid was obtained from RCSB Protein Data Bank with PDB ID: 3DZY [57]. To protonate the structure of protein with Chemistry at HARvard Macromolecular Mechanics (CHARMM) force field [58] and remove crystal water, the crystal structure of RXR protein was prepared by Prepare Protein module in Discovery Studio 2.5 (DS2.5). The binding site for virtual screening was defined by the volume and location of 9-*cis*-retinoic acid. A total of 9,029 nonduplicate TCM compounds from TCM Database@Taiwan [59] were protonating the structure by Prepare Ligand module in DS2.5 after filtering by Lipinski's Rule of Five [60]. The sequence of RXR protein from Swiss-Prot (UniProtKB: P19793) was employed to predict the disordered amino acids using PONDR-Fit [61].

### 2.2. Docking Simulation

The TCM compounds were virtually screened by LigandFit protocol [62] in DS2.5. The LigandFit docking procedure was performed by five major steps. Firstly, it generates candidate ligand conformations using Monte Carlo for docking. Then it positions each ligand conformation in the binding site by multiple orientation or permutation sampling of the ligand principal moments with the principal moments of the site. The docking poses were then optionally minimized with CHARMM force field [58] and calculated the score using the dock score energy function as follows:

(1) Dock Score=−(ligand  receptor  interaction  energy+ligand internal energy).

A pose-saving algorithm was employed to compare the candidate poses and reject the similar poses.

### 2.3. Molecular Dynamics (MD) Simulation

Gromacs [63] is employed to study protein dynamics using classical molecular dynamics theory. The global MD algorithm is defined by four major parts. (1) We input the initial conditions with potential interaction (*V*), position (*r*), and velocities (*v*) for all atoms in the system in this part. (2) The program computes the forces on each atom in the system as follows:

(2) $$\begin{array}{c} F_{i}=\frac{\partial V}{\partial r_{i}}, \end{array}$$

where *F*_*i*_ is obtained by calculating the forces between nonbonded atom pairs, bonded interactions, and restraining and external forces.

The potential and kinetic energies and the pressure tensor are also computed in this part. (3) The MD program updates the configuration by simulating the movement of the atoms using Newton's equations of motion as follows:

(3) $$\begin{array}{c} \frac{\partial r_{i}}{\partial t}=v_{i};\;\;\frac{\partial v_{i}}{\partial t}=\frac{F_{i}}{m_{i}}. \end{array}$$

(4) The MD program outputs the information of positions, velocities, energies, temperature, pressure, and so forth. Finally, the MD program repeats the 2–4 parts for the required number of steps.

The molecular dynamics simulations (MD) are performed by Gromacs. To obtain the initial conditions of each protein-ligand complex, the topology of RXR protein, including charmm27 force fields, was reprepared by Gromacs. The topology and parameters of each ligand for use with Gromacs were provided by SwissParam program [64]. The program has employed a cubic box with a minimum distance of 12 Å from the molecules periphery and solvated by a water model of TIP3P. Firstly, a maximum of 5,000 steps energy minimization were performed using Steepest Descent algorithm [65]. Then a single constant temperature (NVT ensemble) equilibration was performed using Berendsen weak thermal coupling method. The MD program repeats 2–4 parts in a time step unit of 2 fs under the particle mesh Ewald (PME) option to obtain a total of 20 ns production simulation. The 20 ns MD trajectories were analyzed by a series of protocols in Gromacs.

## 3. Results and Discussion

### 3.1. Disordered Protein Prediction

**Figure 1:**
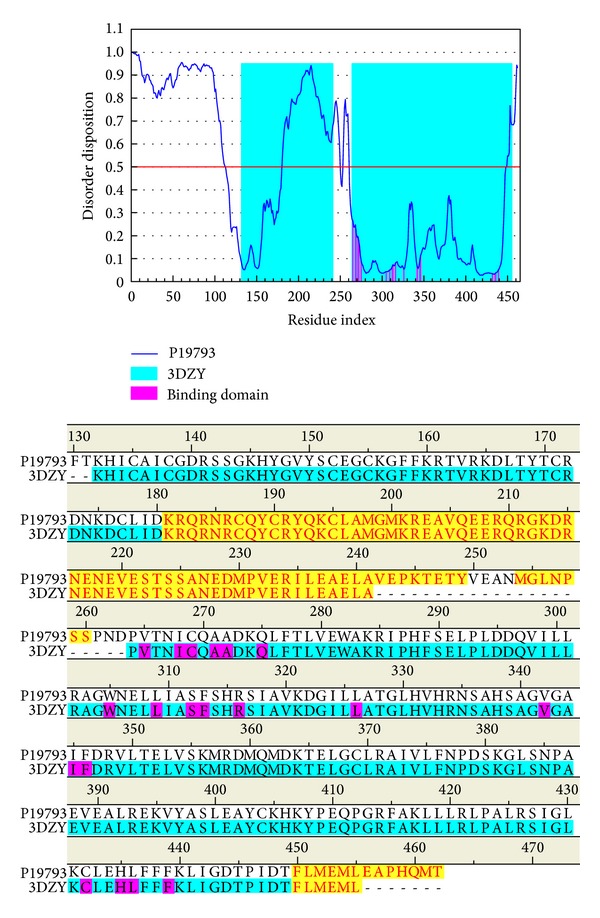
Disordered amino acids predicted by PONDR-Fit and sequence alignment with disordered residues (yellow regions) and residues in the binding domain (magenta regions).

The disordered amino acids of RXR protein were predicted by PONDR-Fit with the protein sequence from Swiss-Prot (UniProtKB: P19793). The sequence alignment and result of disordered amino acids prediction were displayed in Figure 1. The residues in the binding domain do not deposit in the disordered region. It has shown that the RXR protein may have a stable structure of binding domain in protein folding.

### 3.2. Docking Simulation

**Figure 2:**
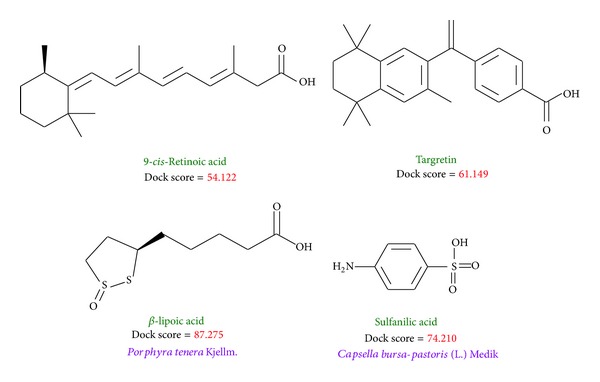
Chemical scaffold of controls and two TCM candidates with their scoring function and sources.

**Figure 3:**
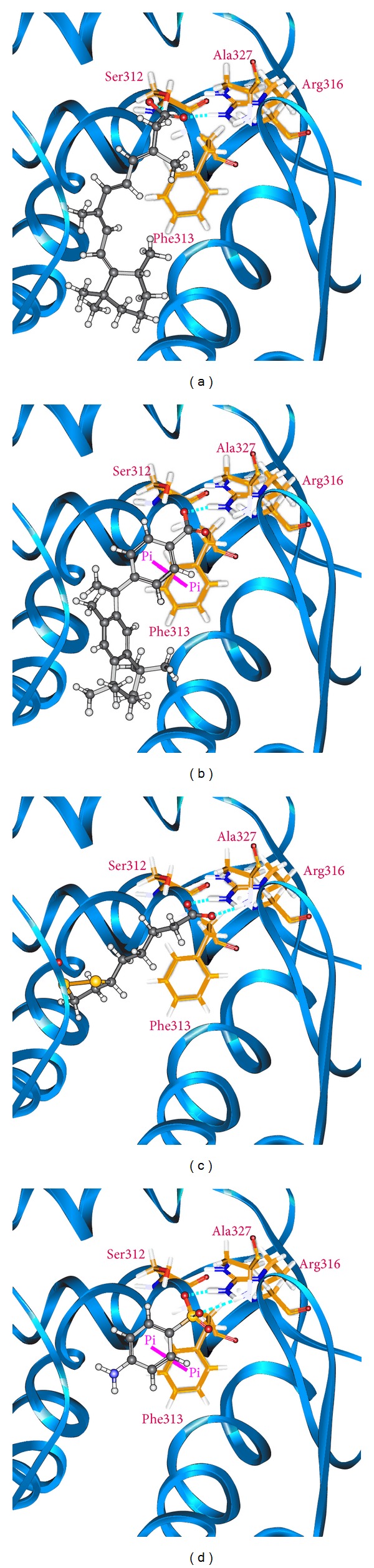
Docking pose of RXR protein complexes with (a) 9-*cis*-retinoic acid, (b) Targretin, (c) *β*-lipoic acid, and (d) sulfanilic acid.

Figure 2 displays the chemical scaffold of 9-*cis*-retinoic acid, Targretin, and the top TCM compounds ranked by dock score with their scoring function and sources. The scoring function of dock score indicates that the TCM compounds *β*-lipoic acid and sulfanilic acid have higher binding affinities than both 9-*cis*-retinoic acid and Targretin. The TCM compound *β*-lipoic acid is extracted from* Porphyra tenera* Kjellm., whereas sulfanilic acid is extracted from* Capsella bursa-pastoris* (L.) Medik. The docking pose of RXR protein complexes with 9-*cis*-retinoic acid, Targretin, and two top TCM candidates was illustrated in Figure 3. All compounds have a hydrogen bond (H-bond) with key residue Arg316. Moreover, both TCM candidates, *β*-lipoic acid and sulfanilic acid, has a H-bond with residue Ala327. Both Targretin and sulfanilic acid, which have a phenyl group, have a *π* interaction with residue Phe313. It shows that the carboxyl group or the group of sulfonyl hydroxide can form a H-bond with key residue Arg316 in the docking pose. The phenyl group next to the carboxyl group or the group of sulfonyl hydroxide can form a *π* interaction with residue Phe313.

### 3.3. Molecular Dynamics Simulation

**Figure 4:**
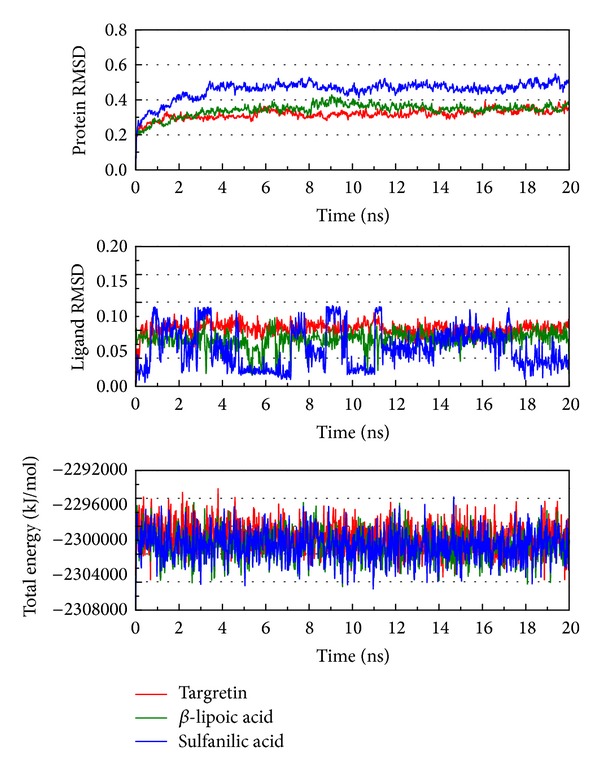
Root-mean-square deviations in units of nm and total energies over 20 ns MD simulation for RXR protein complexes with Targretin, *β*-lipoic acid, and sulfanilic acid.

**Figure 5:**
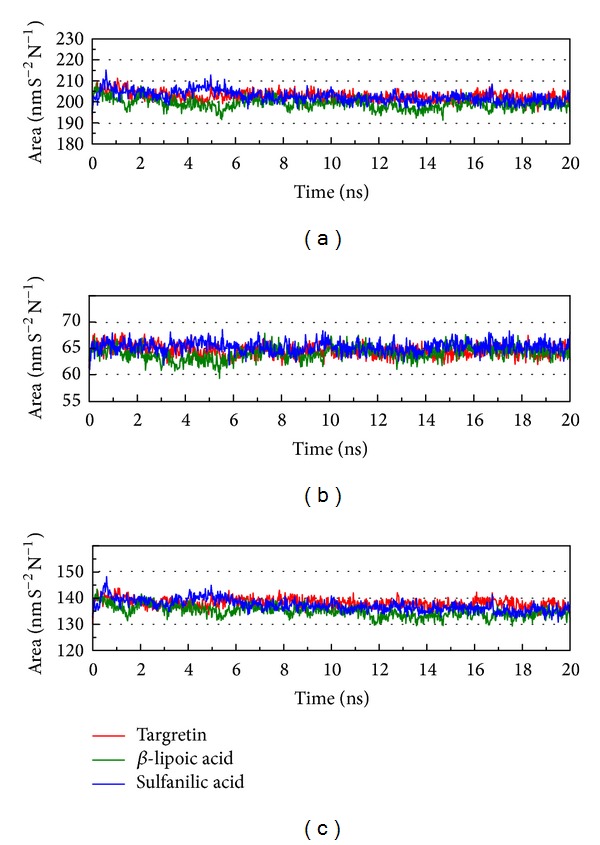
Variation of (a) total solvent accessible surface area, (b) hydrophobic surface area, and (c) hydrophilic surface area over 20 ns MD simulation for RXR protein complexes with Targretin, *β*-lipoic acid, and sulfanilic acid.

**Figure 6:**
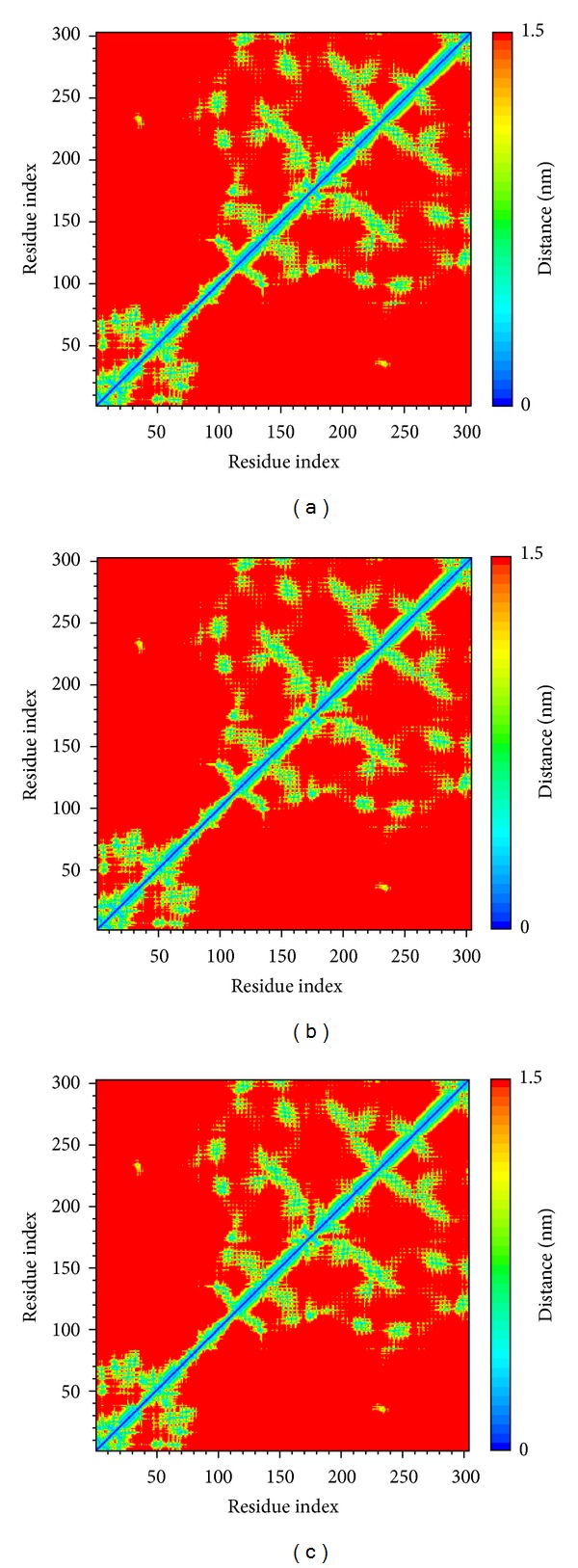
Distance matrices consisting of the mean smallest distance between residue pairs for RXR protein complexes with (a) Targretin, (b) *β*-lipoic acid, and (c) sulfanilic acid. Residues 1–110 in *y*-axis correspond to residues 132–241. Residues 111–302 in *y*-axis correspond to residues 264–455.

As the docking simulations are perform in the condition of rigid body of RXR protein, the molecular dynamics (MD) simulations were then employed to study of protein dynamics and analyze the stability of interactions for each docking poses. The root-mean-square deviations (RMSDs) and total energies over 20 ns MD simulation for RXR protein complexes with Targretin, *β*-lipoic acid, and sulfanilic acid are illustrated in Figure 4. The complexes with Targretin, *β*-lipoic acid, and sulfanilic acid tend to be stable after 16 ns, 16.2 ns, and 17.4 ns MD simulation, respectively. The analysis of solvent accessible surface area for complexes under dynamics condition in Figure 5 indicates that the RXR protein complexes with Targretin, *β*-lipoic acid, and sulfanilic acid have similar hydrophobic and hydrophilic surface area and the MD simulation tends to be stable.  Figure 6 also shows that the RXR protein has similar mean smallest distance between residue pairs for protein complex with Targretin and two top TCM compounds. They indicate that the protein structure of RXR protein complex with top two TCM compounds, *β*-lipoic acid and sulfanilic acid, may not cause the significant differences from docking with Targretin.

**Figure 7:**
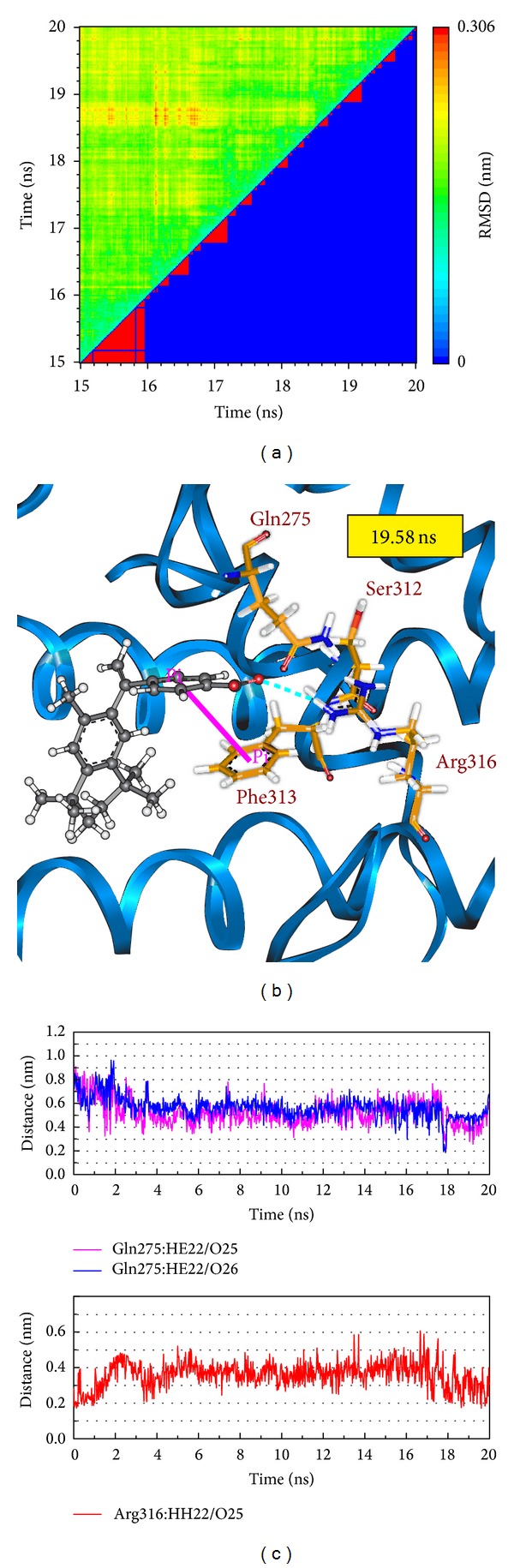
(a) Root-mean-square deviation value (upper left half) and graphical depiction of the clusters with cutoff 0.11 nm (lower right half) for RXR protein complexes with Targretin. (b) Docking poses of middle RMSD structure in the major cluster for RXR protein complexes with Targretin. (c) Distances of hydrogen bonds with common residues during 20 ns MD simulation.

**Figure 8:**
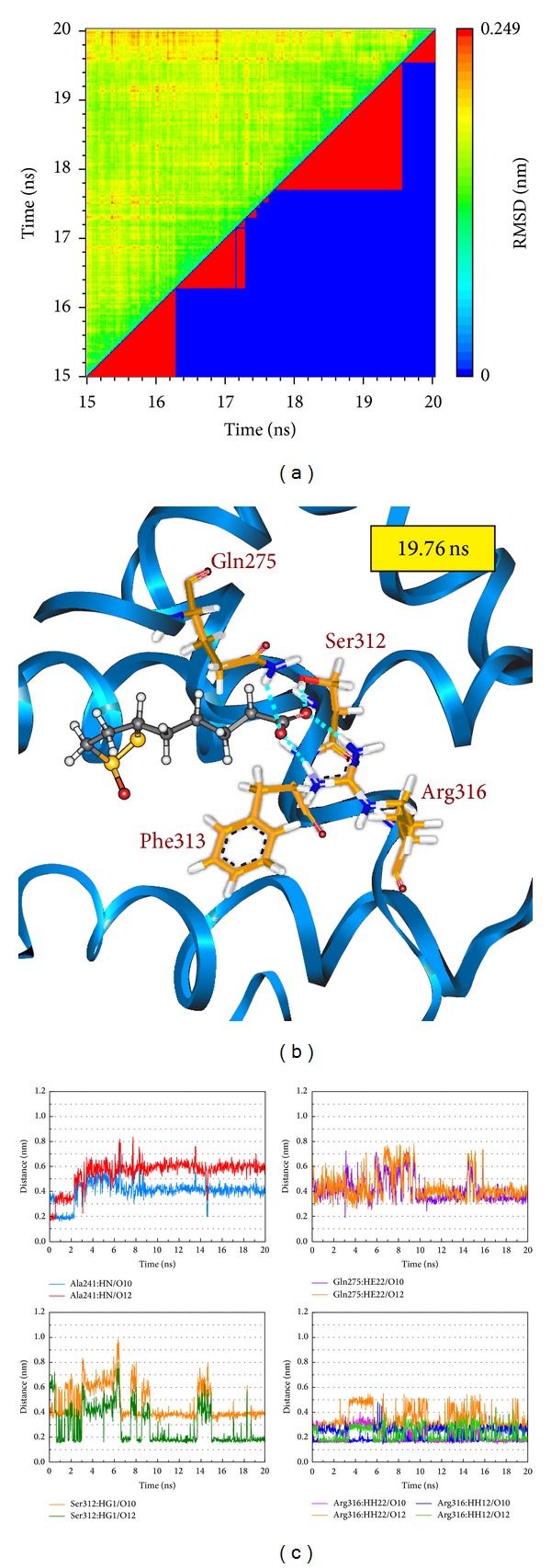
(a) Root-mean-square deviation value (upper left half) and graphical depiction of the clusters with cutoff 0.11 nm (lower right half) for RXR protein complexes with *β*-lipoic acid. (b) Docking poses of middle RMSD structure in the major cluster for RXR protein complexes with *β*-lipoic acid. (c) Distances of hydrogen bonds with common residues during 20 ns MD simulation.

**Figure 9:**
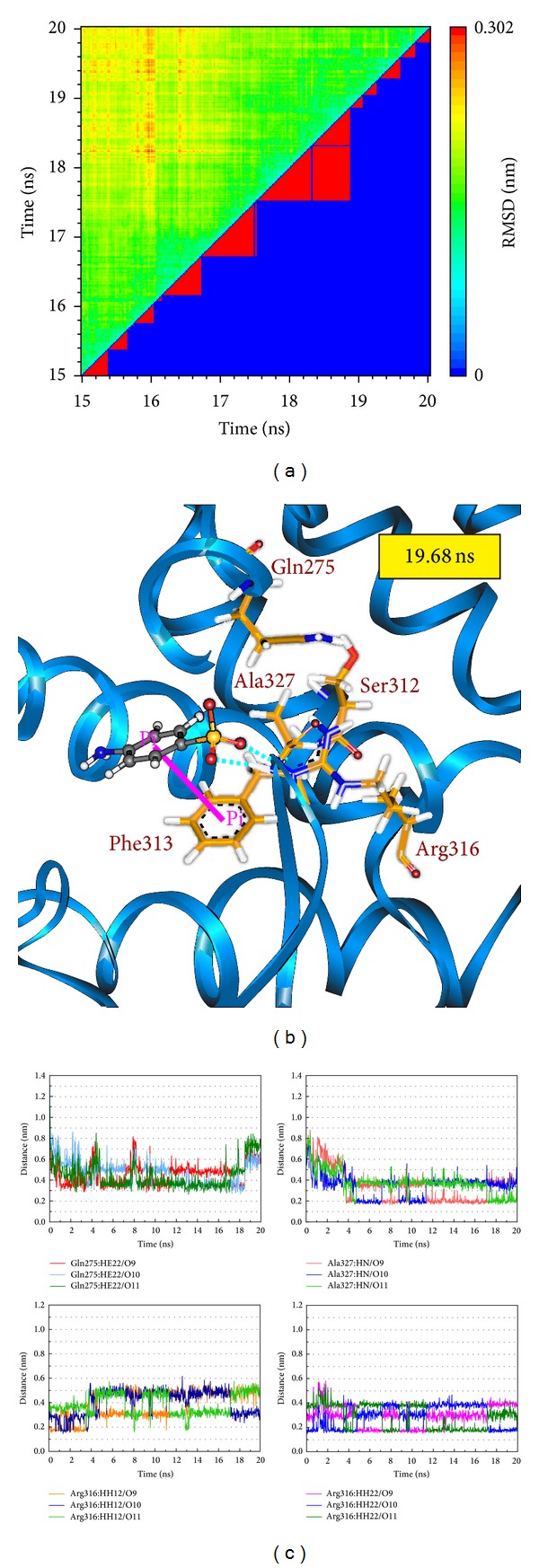
(a) Root-mean-square deviation value (upper left half) and graphical depiction of the clusters with cutoff 0.11 nm (lower right half) for RXR protein complexes with sulfanilic acid. (b) Docking poses of middle RMSD structure in the major cluster for RXR protein complexes with sulfanilic acid. (c) Distances of hydrogen bonds with common residues during 20 ns MD simulation.

**Figure 10:**
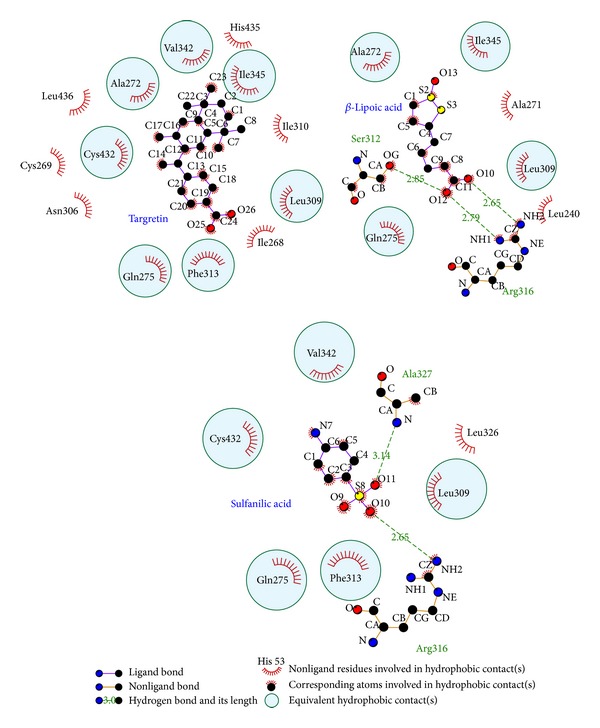
Docking poses of middle RMSD structure in the major cluster for RXR protein complexes drawn by LIGPLOT program.

Root-mean-square deviation value and graphical depiction of the clusters with cutoff 0.11 nm for each RXR protein complexes are employed to display the RMSD values between MD trajectories and identify the middle RMSD structure in the major cluster as the representative structures of each protein-ligand complex after MD simulation. For RXR protein complexes with Targretin, the docking poses of middle RMSD structure in the major cluster after 16 ns MD simulation are illustrated in Figure 7(b). Targretin has the similar docking pose as docking simulation and maintains the H-bond with key residue Arg316 and *π* interaction with residue Phe313. According to the occupancies of H-bonds for common residues of RXR protein listed in Table 1 and the fluctuation of distances for H-bonds displayed in Figure 7(c), they show that the carboxyl group of Targretin can form stable interactions with residue Arg316. For RXR protein complexes with *β*-lipoic acid, the representative structures of docking pose after MD simulation are illustrated in Figure 8(b). *β*-lipoic acid maintains the H-bond with key residue Arg316 and forms the other H-bonds with residue Gln275 and Ser313, which can stabilize the docking pose in the binding domain. From the occupancies of H-bonds for common residues of RXR protein listed in Table 1 and the fluctuation of distances for H-bonds displayed in Figure 8(c), they indicate that the H-bonds between *β*-lipoic acid and residue Ala241 existing in the initial period of MD simulation were not stable. However, the carboxyl group of *β*-lipoic acid has stable H-bonds with residues Arg316, Gln275, and Ser313. From the representative structures of RXR protein complex with sulfanilic acid after MD simulation displayed in Figure 9(b), it is illustrated that the group of sulfonyl hydroxide of sulfanilic acid maintains the H-bonds with key residues Arg316 and Ala327 and the phenyl group next to sulfonyl hydroxide keeps the *π* interaction with residue Phe313. The fluctuation of distances for H-bonds displayed in Figure 9(c) indicates that sulfanilic acid had the H-bonds with residue Gln275, but it misses those H-bonds when the system tends to be stable after 18 ns MD simulation.  Figure 10 displays the docking poses of the representative structures for RXR protein complexes with Targretin, *β*-lipoic acid, and sulfanilic acid. It indicates that the TCM candidates, *β*-lipoic acid and sulfanilic acid, have some equivalent hydrophobic contacts with residues Ala272, Gln275, Leu309, Phe313, Val342, Ile345, and Cys432 as Targretin. These hydrophobic contacts hold the compounds in the binding domain.

## 4. Conclusion

The cholesterol transport protein apolipoprotein E plays the important role in the clearance of amyloid-*β* from the brain, and the transcription of apolipoprotein E expression is also regulated by the heterodimers of RXR with PPAR-*γ* and LXRs. In this study, we aim to investigate the potent TCM candidates of agonists for RXR protein, and the prediction of disordered amino acids of RXR protein was performed to discuss the stability of residues for RXR protein before virtual screening. The top TCM candidates, *β*-lipoic acid and sulfanilic acid, had higher potent binding affinities than both 9-*cis*-retinoic acid and Targretin. After optimizing the result of docking simulation to validate the stability of H-bonds between each ligand and RXR protein under dynamic conditions, the top TCM compounds, *β*-lipoic acid and sulfanilic acid, have stable H-bonds with residues Arg316 and some equivalent hydrophobic contacts with residues Ala272, Gln275, Leu309, Phe313, Val342, Ile345, and Cys432 as Targretin. The carboxyl group or the group of sulfonyl hydroxide can form a H-bond with key residue Arg316 in the docking pose, and the phenyl group next to the carboxyl group or the group of sulfonyl hydroxide can form a *π* interaction with residue Phe313. In addition, *β*-lipoic acid has stable H-bonds with residues Gln275 and Ser313, and sulfanilic acid has stable H-bonds with residue Ala327. These stable H-bonds may strengthen and stabilize TCM candidates inside the binding domain of RXR protein. Hence, we propose *β*-lipoic acid and sulfanilic acid as potential lead compounds for further study in drug development process with the RXR protein against Alzheimer's disease.

## Figures and Tables

**Table 1:** H-bond occupancy for key residues of RXR protein with Targretin and two candidates overall 20 ns molecular dynamics simulation.

Name	H-bond Interaction	Occupancy
Targretin	Gln275:HE22/O25	1%
	Gln275:HE22/O26	1%
	Arg316:HH22/O25	17%

*β*-lipoic acid	Ala241:HN/O10	10%
	Ala241:HN/O12	3%
	Gln275:HE22/O10	2%
	Gln275:HE22/O12	2%
	Ser312:HG1/O12	60%
	Arg316:HH22/O10	89%
	Arg316:HH22/O12	39%
	Arg316:HH12/O10	96%
	Arg316:HH12/O12	86%

Sulfanilic acid	Gln275:HE22/O9	1%
	Gln275:HE22/O10	1%
	Gln275:HE22/O11	2%
	Arg316:HH22/O9	53%
	Arg316:HH22/O10	45%
	Arg316:HH22/O11	49%
	Arg316:HH12/O9	25%
	Arg316:HH12/O10	23%
	Arg316:HH12/O11	16%
	Ala327:HN/O9	39%
	Ala327:HN/O10	24%
	Ala327:HN/O11	17%

H-bond occupancy cutoff: 0.3 nm.
